# KRAS-elicited PDIA6 blocks PERK-dependent immunogenic cell death in pancreatic carcinoma

**DOI:** 10.1080/2162402X.2026.2641424

**Published:** 2026-03-08

**Authors:** Liwei Zhao, Peng Liu, Guido Kroemer, Oliver Kepp

**Affiliations:** a Institut Universitaire de France, Sorbonne Université, Inserm, Centre de Recherche des Cordeliers, Equipe Labellisée Par La Ligue Contre Le Cancer, Université Paris Cité, Paris, France; b Institut Gustave Roussy, INSERM US23/CNRS UAR 3655, Metabolomics and Cell Biology Platforms, Université Paris-Saclay, Villejuif, France; c Department of Biology, Institut du Cancer Paris CARPEM, Hôpital Européen Georges Pompidou, Paris, France

**Keywords:** Cancer immunotherapy, cell stress, immune exclusion, small molecule

## Abstract

KRAS-mutant pancreatic ductal adenocarcinoma (PDAC) remains largely refractory to immune checkpoint blockade. Wang et al. identified PDIA6 as a KRAS-driven suppressor of PERK-dependent immunogenic cell death, fostering immune exclusion. PDIA6 inhibition restores CD8^+^ T-cell immunity and sensitizes PDAC to immunotherapy, highlighting a targetable resistance mechanism in KRAS-mutant PDAC.

Pancreatic ductal adenocarcinoma (PDAC) is highly immunotherapy-refractory, and its resistance to immune checkpoint inhibitors (ICI) cannot be explained solely by low neoantigen load and stromal exclusion of CD8⁺ cytotoxic T lymphocytes (CTLs).^1^ Accumulating findings suggest that oncogenic programs can actively suppress anticancer immunity in PDAC by blunting dendritic cell (DC) activation and effective T-cell priming, thus contributing to the failure of immunotherapy.^2^ In this context, immunogenic cell death (ICD) has become of central interest, as it facilitates the coordinated emission of danger-associated molecular patterns (DAMPs) that act on DCs and license antigen-presenting cells to prime CTLs.^3^ Thus, the immunologic consequences of cytotoxic stress and tumor cell death depend on whether cancer cells undergo bona fide ICD rather than receding into an immunologically silent quietus. Since PDAC is under poor immunosurveillance, meaning that it usually occurs in the context of the absence of efficient immune infiltration and thus exhibits primary resistance to any tentative of immunotherapy, it can be suspected that ICD is poorly operative in PDAC as well.

ICD is not a passive byproduct of cell death but an active, tightly coordinated stress‒response program that culminates in the emission of immunogenic hallmarks. Central to this process is endoplasmic reticulum (ER) stress signaling through eukaryotic translation initiation factor 2 alpha kinase 3 (EIF2AK3, best known as PERK)-mediated phosphorylation of eukaryotic translation initiation factor 2 subunit alpha (EIF2AS1, best known as eIF2α), which defines the so-called “integrated stress response” (ISR) and also constitutes a biochemical hallmark of ICD.^4^^,^^5^ Activation of the PERK-eIF2α axis not only modulates translation but is required for key downstream events in ICD signaling, including the pre-apoptotic exposure of calreticulin (CALR) on the cell surface.^6^ Surface CALR functions as an “eat-me” signal that promotes dendritic cell (DC)-mediated phagocytosis and depends on the ER stress-driven co-translocation of protein disulfide isomerase family A member 3 (PDIA3, best known as ERp57), a thiol oxidoreductase that complexes with CALR in the ER and on the cell surface. Disruption of the CALR‒PDIA3 interaction abolishes their surface exposure and compromises immunogenicity.^7^ Another defining feature of ICD that is largely controlled by eIF2α phosphorylation consists of the autophagy-dependent release of adenosine triphosphate (ATP) from stressed and dying cancer cells.^7^ Extracellular ATP acts as a purinergic receptor P2Y2-dependent chemoattractant to facilitate the recruitment of DCs into the tumor bed.^8^ In parallel, dying cells release high-mobility group box 1 (HMGB1), which ligates Toll-like receptor 4 (TLR4) on DCs to enhance myeloid differentiation primary response 88 (MYD88)-mediated DC maturation for optimal antigen processing and cross-presentation.^9^ Moreover, ICD can elicit the secretion of type I interferons by cancer cells, a program that often involves nucleic acid sensing by cyclic GMP‒AMP synthase (CGAS) and stimulator of interferon response CGAMP interactor 1 (STING1). Autocrine or paracrine interferon signaling then affects both malignant and myeloid cells^7^ to promote DC maturation, reinforce antigen presentation, induce chemokines such as C-X-C motif chemokine ligand 10 (CXCL10) and sustain CD8⁺ T-cell expansion, collectively ensuring that cellular stress is translated into durable adaptive immunity.^7^

Tumors that evolve under chronic proteotoxic pressure, such as the KRAS proto-oncogene, GTPase (KRAS)-mutant PDAC, rarely undergo immunogenic conversion, suggesting that cancer cells actively dampen stress signaling to prevent crossing the immunogenic threshold.^2^ In this context, the tight regulation of PERK activity represents a strategic immune-evasion mechanism. Yet, the molecular brakes that restrain PERK-dependent ICD in PDAC have remained elusive.

Building on the premise that ER stress-induced immunogenicity is actively suppressed in PDAC, Wang et al. systematically searched for tumor cell-intrinsic regulators associated with CD8⁺ T-cell exclusion and poor prognosis in pancreatic ductal adenocarcinoma.^10^ Through multi-omics intersection analyses of PDAC datasets stratified by CD8⁺ T-cell infiltration and survival, the authors identified protein disulfide isomerase family A member 6 (PDIA6) as a candidate immune suppressor. PDIA6 expression was elevated in tumors compared to adjacent normal tissue, correlated with advanced clinical stage, as well as with reduced intratumoral CD8⁺ T-cell density, and predicted inferior survival. Importantly, in independent PDAC cohorts treated with ICIs, high PDIA6 expression was associated with a poor therapeutic response, positioning PDIA6 as both a mechanistic regulator and a potential predictive biomarker.^10^

Functionally, PDIA6 depletion did not affect the proliferation of PDAC cells *in vitro*, but markedly suppressed tumor growth in immunocompetent mice.^10^ This growth inhibition was abrogated by CD8⁺ T-cell depletion, which established PDIA6 as an immune modulator rather than a cancer cell-intrinsic inhibitor of tumor progression. PDAC tumors in which PDIA6 expression was silenced exhibited increased CD8⁺ T-cell infiltration, enhanced T-cell proliferation, effector cytokine production and augmented dendritic cell activation, which was consistent with the restoration of ICD. Furthermore, transcriptome analyses revealed an enrichment of ICD-related pathways in PDIA6-low tumors. Thus, upon oxaliplatin treatment, PDIA6-deficient cells exhibited enhanced CALR surface exposure, increased autophagy-dependent ATP release and greater dendritic cell phagocytosis and maturation. Vaccination assays further confirmed that PDIA6 knockdown augmented the immunogenicity of dying tumor cells in vivo.^10^

At the molecular level, the study by Wang et al. shows that PDIA6 directly interacts with PERK to inhibit its kinase activity.^10^ Using disulfide-trap mutants and co-immunoprecipitation assays, the authors showed that PDIA6 binds to cysteine 453 within the luminal domain of PERK. This interaction disrupts the formation of intermolecular disulfide bonds required for PERK dimerization and autophosphorylation. As a consequence, PDIA6 attenuates the activation of PERK-mediated eIF2α phosphorylation and downstream ICD signaling. Consistently, mutation of PERK cysteine 453 to serine impaired dimerization and reduced kinase activity, supporting the importance of this redox-sensitive residue in mediating PERK pathway activation. Pharmacologic inhibition of PERK partially reversed the enhanced ICD phenotype observed with PDIA6 loss, further establishing that PDIA6 acts upstream of PERK to regulate the immunogenic threshold.^10^

Importantly, Wang et al. demonstrated that oncogenic KRAS^G12D^ drives PDIA6 transcription via the transcription factor YY1.^10^ KRAS inhibition with MRTX1133 reduced YY1 and PDIA6 expression, and chromatin immunoprecipitation confirmed direct YY1 binding to the PDIA6 promoter. Thus, PDIA6 emerges as a downstream effector of KRAS signaling, linking oncogenic proteotoxic stress to immune evasion. The authors also describe PACMA31, a small-molecule inhibitor that suppresses the disulfide reductase activity of PDIA6. Consistently, PACMA31 augmented PERK-mediated eIF2α phosphorylation, in line with the relief of PDIA6-dependent repression. In orthotopic PDAC models, PACMA31 phenocopied PDIA6 knockdown and suppressed tumor growth in an immune-dependent manner. Notably, PD-1 blockade alone had minimal activity, but combination therapy with PACMA31 significantly reduced the tumor burden. When combined with oxaliplatin to enhance ICD induction, PACMA31 further potentiated the outcome of PD-1 blockade, leading to increased CD8⁺ T-cell infiltration, higher granzyme B expression, enhanced dendritic cell maturation, reduced stromal fibrosis and prolonged survival of KPC mice harboring KRAS mutations.^10^ (Figure 1)

**Figure 1.**
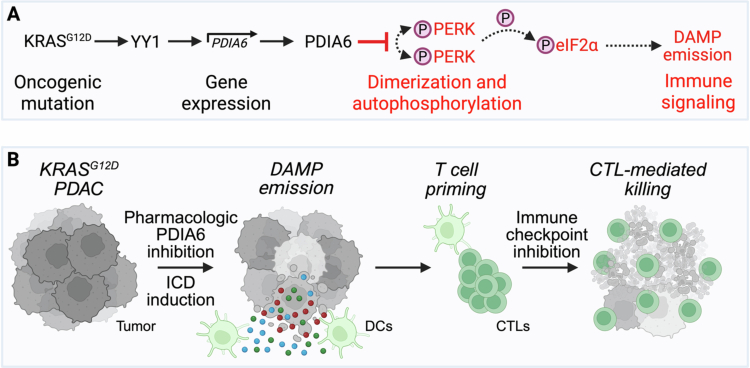
PDIA6 functions as a checkpoint that suppresses PERK-dependent immunogenic cell death in KRAS-mutant PDAC. (A) Oncogenic KRAS^G12D^ upregulates PDIA6 transcription through YY1, increasing PDIA6 expression in pancreatic ductal adenocarcinoma (PDAC) cells. PDIA6, a protein disulfide isomerase, directly interacts with cysteine 453 within the luminal domain of PERK and disrupts the intermolecular disulfide bond formation required for PERK dimerization and autophosphorylation. As a consequence, PDIA6 attenuates the activation of the PERK-eIF2α signaling axis, thereby limiting ER stress-dependent immunogenic cell death (ICD). Suppression of PERK signaling reduces danger-associated molecular pattern (DAMP) emission, resulting in impaired dendritic cell (DC) recruitment and maturation, defective CD8⁺ T-cell priming, and immune exclusion. (B) Pharmacologic inhibition of PDIA6 (e.g., by PACMA31) restores PERK activation, enhances DAMP emission, promotes DC activation, and CD8⁺ T-cell infiltration, and sensitizes PDAC to ICD induction and immune checkpoint blockade. Thus, PDIA6 operates upstream of immune checkpoints as a tumor-intrinsic stress buffer linking oncogenic KRAS signaling to immune resistance.

Altogether, this elegant study delineates a KRAS^G12D^-YY1-PDIA6 signaling axis that attenuates PERK-dependent ER stress responses, thereby suppressing ICD and sustaining immune exclusion in PDAC. Pharmacologic inhibition of PDIA6 restores stress signaling, sensitizes tumors to chemotherapy-induced ICD, and renders them responsive to immune checkpoint blockade. These findings position PDIA6 as a redox-regulated proteostasis node acting upstream of canonical checkpoints and offer a compelling strategy to overcome immunotherapy resistance in KRAS-mutant PDAC. More broadly, this study highlights proteostasis control as a determinant of whether oncogenic stress culminates in immune activation or immune escape, thus opening a new conceptual layer for cancer immunotherapy.

## Data Availability

Data sharing is not applicable to this article, as no new data were created or analyzed in this study.
